# DenseViT-OCT: A Hybrid CNN-Transformer Architecture with Multi-Scale Dense Feature Aggregation for Automated Epiretinal Membrane Severity Classification

**DOI:** 10.3390/tomography12060076

**Published:** 2026-05-22

**Authors:** Elif Yusufoğlu, Salih Taha Alperen Özçelik, Orhan Atila, Numan Halit Guldemir, Abdulkadir Sengur

**Affiliations:** 1 Department of Ophthalmology, Elazig Fethi Sekin City Hospital, 23100 Elazig, Turkey; elif.eraslan@yahoo.com; 2 Department of Electrical-Electronics Engineering, Faculty of Engineering, Bingol University, 12000 Bingol, Turkey; sozcelik@bingol.edu.tr; 3 Department of Electrical-Electronics Engineering, Faculty of Technology, Firat University, 23100 Elazig, Turkey; ksengur@firat.edu.tr; 4 School of Electronics, Electrical Engineering and Computer Science, Queen’s University Belfast, Belfast BT9 5BN, UK; nguldemir01@qub.ac.uk

**Keywords:** epiretinal membrane, optical coherence tomography, deep learning, hybrid CNN-Transformer, vision transformer, medical image classification, computer-aided diagnosis, retinal imaging, multi-scale feature fusion, attention mechanism

## Abstract

Background/Objectives: Epiretinal membrane (ERM) is a common vitreoretinal disorder characterized by fibrocellular proliferation on the inner retinal surface, often leading to progressive visual impairment. Accurate grading of ERM severity using optical coherence tomography (OCT) is critical for treatment planning and surgical decision-making; however, manual grading is labor-intensive and subjective. This study aims to develop an automated and reliable deep learning-based method for ERM severity classification. Methods: We propose DenseViT-OCT, a hybrid deep learning model that integrates dense convolutional neural networks (CNN) and vision transformers (ViT). The model introduces three key modules: Multi-Scale Dense Feature Aggregation (MDFA) for capturing hierarchical features across multiple spatial scales, Adaptive Feature Calibration (AFC) for enhancing feature discrimination through channel and spatial attention, and Cross-Attention Feature Fusion (CAFF) for enabling bidirectional interaction between convolutional and transformer representations. The model was trained and evaluated on 2195 OCT B-scan images obtained from 397 patients. Results: DenseViT-OCT achieved an overall accuracy of 94.76% on the internal four-class test set, outperforming 19 benchmark models, including ConvNeXt, EfficientNet, ViT, and Swin Transformers. The model demonstrated balanced performance with a macro-averaged precision of 93.76%, recall of 93.22%, F1-score of 93.47%, Cohen’s kappa of 92.62%, and macro-Area Under the Curve (AUC) of 98.95%. Ablation experiments confirmed the contribution of the proposed MDFA, AFC, CAFF, and deep supervision components, with the full model consistently outperforming reduced variants and standalone DenseNet121 and ViT-B/16 backbones. In repeated experiments across five random seeds, DenseViT-OCT also achieved the best mean accuracy (0.9399 ± 0.0052). External validation on the public multicenter OCTDL dataset, performed as binary ERM-versus-normal classification because of label availability, yielded 90.76% accuracy and 97.61% AUC, indicating promising generalization beyond the development cohort. Conclusions: DenseViT-OCT provides a robust framework for automated ERM severity classification from OCT B-scans. The combination of local CNN features, global transformer context, and dedicated fusion modules improves classification performance and yields clinically meaningful error patterns. Although further stage-wise multicenter validation, volumetric OCT analysis, and prospective clinical assessment are required, the proposed method shows promise as a research-oriented decision-support framework for B-scan-level ERM assessment.

## Simple Summary

Epiretinal membrane is a common retinal disease that can gradually impair vision if not treated appropriately. Its severity is usually assessed using optical coherence tomography images, but manual evaluation can be time-consuming and subjective. In this study, we developed a new artificial intelligence model that automatically classifies disease severity. The model combines different deep learning techniques to better capture retinal structures and improve decision accuracy. It achieved high performance and reliably focused on clinically relevant areas of the retina. This approach may support clinicians in diagnosis, improve treatment planning, and encourage further research in automated retinal image analysis.

## 1. Introduction

The epiretinal membrane (ERM) is an eye disease that occurs when the epiretinal membrane adheres to the internal limiting membrane (ILM), most commonly in the macula, causing structural and functional deterioration of the ILM [1]. While there are many factors contributing to the development of ERM, the most prominent is age-related posterior vitreous detachment. Other contributing factors include retinal tears, diabetic retinopathy, vascular occlusion, and complications following retina surgery [2].

Clinically, the age-related progression of ERM formation generally follows a specific pattern. With aging, the vitreous gel begins to shrink and liquefy, eventually leading to posterior vitreous detachment (PVD) [3]. As the vitreous separates from the posterior retina, patients experience floaters, photopsia, or transient visual disturbances. Accumulating with PVD, the tensile forces applied to the retina cause micro damage to the ILM. ERMs develop as a result of glial cells migrating to the ILM surface and proliferating. Therefore, while ERMs are rarely seen in younger individuals, they are becoming increasingly common in individuals over the age of 60–70, particularly in association with PVD. According to studies conducted in different regions, the prevalence of ERM in the population is between 2.2% and 28% [3,4,5].

In the period when retinal imaging techniques were not sufficiently developed, the diagnosis of Epiretinal Membrane (ERM) was largely based on histopathological examinations. During this process, surgically removed membrane samples were evaluated using methods such as light microscopy, immunohistochemical analyses, and electron microscopy to reach a diagnosis [6]. Initially, basic techniques such as fundus photography and fluorescein angiography were used for retinal imaging [7]. Following this period, with the introduction of advanced imaging methods, such as OCT and OCTA, into clinical use, significant progress has been made in the diagnosis and treatment of ERM. These modern techniques have significantly strengthened clinical decision-making processes by enabling high-resolution and layer-by-layer examination of retinal microstructures [8,9].

Progress in retinal imaging technology has been concurrent with and complementary to the diagnostic potential of high-resolution optical coherence tomography (OCT), OCT angiography (OCTA), and intraoperative OCT (iOCT) techniques used in the identification and evaluation of ERM. Reviews suggest that OCT along with its newer variants is an essential component in the diagnosis of ERM, as well as in various other eye diseases, both at the stages of initial diagnosis, preoperative planning, and monitoring postoperatively [2,10]. Specifically, it was observed that biomarkers associated with OCTA, such as abnormality in the foveal avascular zone, and changes in the thickness of retinas, were correlated with the degree of metamorphopsia in patients with idiopathic ERM [11]. The combination of advances in high-resolution retinal imaging and AI-based models led to the automation of ERM detection, severity classification, and segmentation tasks based on OCT scans [12,13].

Meta-analyses using OCT images from different healthcare centers and imaging devices show that when evaluated in terms of performance metrics such as classification accuracy, sensitivity, and AUC, artificial intelligence models achieve results quite close to those of expert clinicians. Furthermore, recent studies reveal that deep neural networks, when used, are not limited to simply detecting the presence of ERMs but are also evolving into advanced decision support systems capable of indicating the size and location of pathological foci through segmentation [14]. In clinical practice, ERM grading influences several key decisions, including whether a patient can be monitored conservatively, whether closer follow-up is required, and whether pars plana vitrectomy with membrane peeling should be considered. Manual grading from OCT B-scans is feasible for experienced retina specialists, but it remains time-consuming, observer-dependent, and difficult to standardize across institutions, especially in borderline cases between adjacent severity stages. Therefore, an automated and interpretable ERM grading system may support routine screening, reduce assessment variability, and provide more consistent severity estimation for treatment planning and longitudinal follow-up. Recent OCT-based studies suggest that ERM severity is not merely an anatomical descriptor but also a clinically meaningful biomarker associated with visual acuity deterioration, metamorphopsia severity, retinal microstructural disruption, and postoperative prognosis. Progressive ERM stages have been linked to increasing retinal distortion, microvascular alterations, and functional decline, highlighting the importance of accurate automated severity grading for treatment planning and surgical decision support [9].

Existing deep learning studies on ERM analysis can be grouped into three main directions. First, some studies have focused on multi-class ERM severity grading, often by combining structural preprocessing or segmentation with classification networks. For example, Jin et al. [15] proposed a two-stage interpretable framework for six-class ERM grading across multiple centers and OCT devices, while Yan et al. [16] developed a joint classification-segmentation system for ERM severity assessment. These studies highlight the clinical importance of stage-wise grading, but they also show that fine-grained ERM classification remains challenging. Second, several works have addressed binary ERM detection or screening, where the task is to distinguish ERM from normal OCT findings rather than to determine severity stage. Gende et al. [17] introduced a multi-task end-to-end model for joint ERM segmentation and binary classification, Mora et al. [18] reported very high performance for ERM detection using transfer learning, and Ayhan et al. [19] explored ensemble learning for multi-class ERM discrimination. Although these studies demonstrate the potential of deep learning for ERM-related analysis, binary or lower-granularity settings are less demanding than detailed severity grading. The authors used the t-distributed Stochastic Neighbor Embedding (t-SNE) approach for explainability analysis. The broader AI-based OCT studies have addressed abnormality detection across multiple retinal diseases and prediction of postoperative visual outcomes, indicating the expanding role of artificial intelligence in ophthalmic imaging and ERM-related decision support [20,21,22]. More broadly, recent medical image analysis studies have also emphasized the value of uncertainty-aware learning strategies and hybrid transformer-based designs, such as adaptive Bayesian iterative learning for cardiac image analysis and multi-axis vision transformer architectures for medical image segmentation, further supporting the importance of robust contextual modeling in clinically oriented imaging tasks [23,24]. Despite this progress, relatively few studies have investigated a dedicated hybrid CNN-transformer design for ERM severity classification that explicitly integrates multi-scale CNN features, adaptive feature recalibration, and bidirectional cross-modal fusion. This gap motivated the development of the proposed DenseViT-OCT framework. A summary of representative studies on ERM classification, including datasets, number of classes, and reported performance metrics, is presented in Table 1. 

AI-assisted ophthalmic disease detection systems that approach expert-level performance may help reduce clinician workload and improve consistency in image-based assessment. The main contributions of this study are threefold. First, we propose DenseViT-OCT, a hybrid CNN-transformer framework that combines DenseNet121 and ViT-B/16 to jointly capture local retinal microstructures and global morphological context for ERM severity grading. Second, we introduce three dedicated feature processing modules—MDFA, AFC, and CAFF—together with a deep supervision strategy to improve multi-scale representation learning and cross-modal fusion. Third, we provide a comprehensive evaluation including comparison with 19 benchmark models, ablation analysis, repeated multi-run statistical analysis, external validation on a public multicenter dataset at the binary ERM-versus-normal level, and explainability assessment using Grad-CAM++ and LIME.

The remainder of this article is structured as follows. Section 2 describes our proposed DenseViT-OCT method, including the dataset, preprocessing, network architecture, novel feature processing modules (MDFA, AFC, CAFF), multi-branch classification strategy, and training details. Section 3 presents all experimental results, including evaluation metrics, performance comparison with 19 baseline models, class-by-class performance, confusion matrix analysis, and visualizations to facilitate understanding of the results. Section 4 discusses why the hybrid architecture design can deliver better performance, its benefits for clinical use, how it can be interpreted, comparisons with similar studies, limitations and considerations, as well as future research directions. Section 5 summarizes our contributions and explains how they can transform automated ERM evaluation.

## 2. Materials and Methods

In this section, we present our novel hybrid deep learning architecture for automated classification of epiretinal membrane (ERM) severity from OCT images. Our approach combines the dense feature propagation capabilities of DenseNet121 with the global context modeling power of ViT, augmented by custom-designed modules for enhanced feature extraction and fusion.

### 2.1. Dataset and Preprocessing

#### 2.1.1. Dataset Composition

This study utilized a retrospective OCT dataset compiled for ERM severity assessment. The dataset consisted of 2195 retinal B-scan OCT images obtained from 397 patients. The study was conducted in accordance with the Declaration of Helsinki and approved by the Firat University Non-Interventional Research Ethics Committee, Session No. 2025/19-27. All images were anonymized prior to analysis.

The dataset was divided into four classes representing increasing ERM severity: Normal, ERM Stage 1 (mild), ERM Stage 2 (moderate), and ERM Stage 3 (advanced/severe). The Normal class contained 920 images from 83 patients (41.91% of the dataset). The ERM Stage 1 class contained 405 images from 151 patients (18.45%), the ERM Stage 2 class contained 445 images from 135 patients (20.27%), and the ERM Stage 3 class contained 425 images from 100 patients (19.36%).

A total of 1119 images were obtained from the right eye and 1076 from the left eye. Among the 397 patients, 98 contributed OCT scans from both eyes, 146 contributed scans only from the right eye, and 153 contributed scans only from the left eye. The number of OCT slices per patient ranged from 1 to 64, with an overall mean of 5.53 slices. The average number of slices per patient was 11.08 in the Normal group and ranged from 2.68 to 4.25 across the ERM groups. This distribution reflects routine clinical practice, where fewer slices may be sufficient once pathology is visually evident.

**Figure 1:**
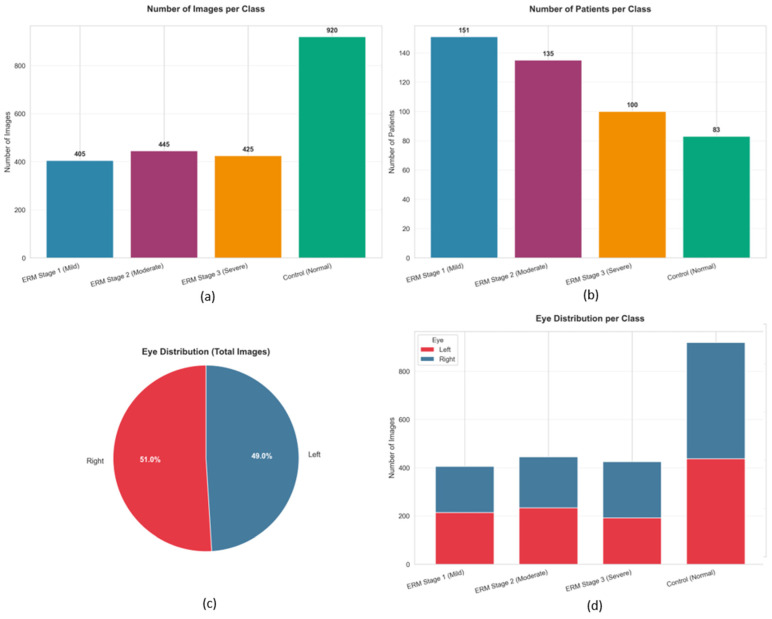
Dataset distribution charts—(**a**) Number of images per class, (**b**) Number of patients per class, (**c**) Eye distribution total, (**d**) Eye distribution per class.

The class-wise laterality distribution was also relatively balanced. The Stage 1 class included 214 left-eye and 191 right-eye images; the Stage 2 class included 233 left-eye and 212 right-eye images; the Stage 3 class included 192 left-eye and 233 right-eye images; and the Normal class included 437 left-eye and 483 right-eye images. Figure 1 summarizes the distribution of images, patients, and eye laterality across the dataset.

Detailed demographic variables such as age and sex were not systematically available for the present retrospective dataset and were therefore not included in the current analysis.

#### 2.1.2. Ground Truth Annotation and Labeling Procedure

Ground-truth labels were assigned by a single ophthalmologist with more than 15 years of clinical experience in retinal disease assessment and OCT interpretation. The labeling process was based on the OCT staging principles described by Govetto et al. [9], and adapted to the four-class categorization used in this study: Normal, ERM Stage 1, ERM Stage 2, and ERM Stage 3.

The stage definitions were determined according to membrane appearance and the degree of structural retinal distortion visible on OCT B-scans. Normal cases showed no epiretinal membrane-related tractional abnormality. Stage 1 (mild ERM) corresponded to thin membrane formation with preserved foveal depression and only mild retinal surface undulations. Stage 2 (moderate ERM) was defined by loss of the foveal pit, more evident retinal surface wrinkling or pucker, and disruption involving the photoreceptor-related layers. Stage 3 (advanced/severe ERM) included dense membrane formation accompanied by marked retinal distortion, retinal thickening, and prominent folds.

Because the dataset was retrospectively labeled by a single expert, inter-observer agreement could not be assessed. This should be considered a limitation of the present study, particularly for borderline cases between adjacent severity stages.

#### 2.1.3. Data Preprocessing and Augmentation

A uniform pipeline was used to preprocess all of the OCT pictures to make sure they were all the same and to make the model more general. We resized the images to 224 × 224 pixels to fit the input requirements of both DenseNet121 and Vision Transformer architectures [25]. First, we normalized the pixel values to the interval [0, 1] and then standardized the images using the statistics of the ImageNet dataset (mean = [0.485, 0.456, 0.406], std = [0.229, 0.224, 0.225]) to utilize the power of transfer learning. To reduce the risk of overfitting and maintain the stability of the model, we used a variety of geometric and photometric transformations during the training process. These transformations include random horizontal flip with a probability of 0.5, random rotation of ±15°, random changes in brightness and contrast of ±20%, Gaussian noise with a sigma value of 0.01, and small random affine transformations with a shift of ±10%. To prevent data leakage, stratified splitting was performed at the patient level. This ensures that all images of a patient remain in the same set: training, validation, or test. The data was then split into 60% for training (238 patients), 20% for validation (79 patients), and 20% for testing (80 patients). The careful preprocessing of the data and the use of data augmentation techniques, where the data remains separate at the patient level, not only help the model to memorize the scanning patterns but also generalize the performance of the model to other unseen patients [26].

### 2.2. Network Architecture Overview

**Figure 2:**
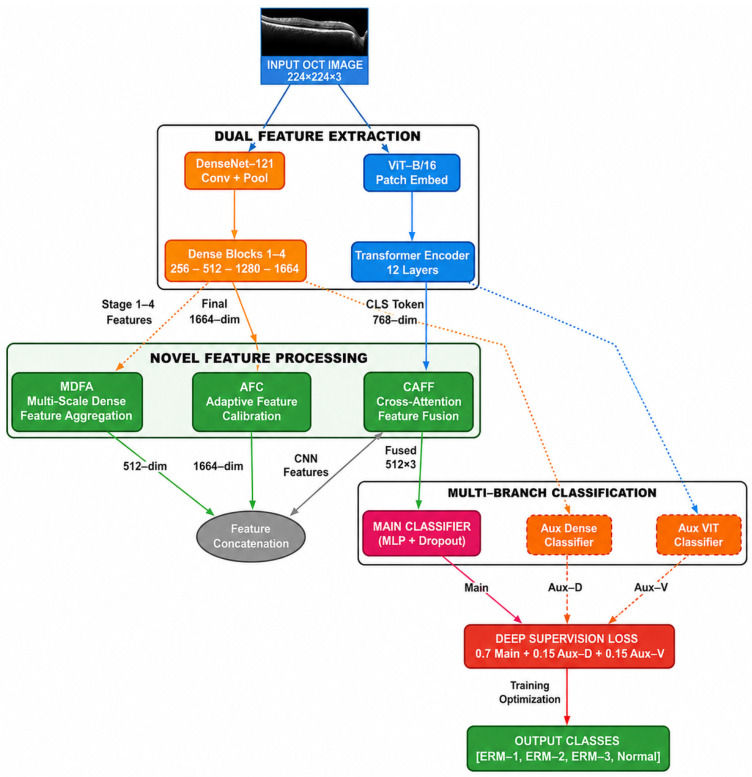
Overall architecture diagram of the proposed DenseViT-OCT model. CNN denotes convolutional neural network, ViT denotes Vision Transformer, and Aux denotes auxiliary classifier.

The architecture we propose is called DenseViT-OCT and uses a two-stream design with convolution-based and transformer-based feature extraction. As shown in Figure 2, the model consists of five main components. DenseNet121 [27] backbone is used for hierarchical local feature extraction. Vision Transformer ViT-B/16 branch, which operates via patch embedding, is included for global context modeling [28]. In addition, there are three new feature processing modules: MDFA, AFC and CAFF. A multi-branch classification head with deep supervision is used for classification. A weighted loss function is applied for training optimization. The rationale for this hybrid design is grounded in the complementary characteristics of OCT interpretation. CNNs are well suited to detecting fine-grained local retinal changes, such as membrane boundaries, layer irregularities, and focal distortions, whereas transformers are better able to model long-range contextual dependencies across the full B-scan. ERM severity assessment requires both levels of information simultaneously; therefore, a hybrid architecture is particularly appropriate for this task.

Given an input OCT image $X\in R^{H\times W\times 3}$ where H=W=224, the image is simultaneously processed through both the CNN and transformer pathways. The DenseNet-121 backbone network extracts the multi-scale hierarchical features through four dense blocks, where the spatial sizes of the feature maps become smaller while the semantic information becomes more complex. At the same time, the ViT branch processes the input image into patches of size 16 × 16, followed by 12 transformer encoder layers to capture the spatial information. Then, the features are refined by our custom network and finally fused together for the final classification.

### 2.3. DenseNet121 Backbone for Hierarchical Feature Extraction

We use DenseNet121 as the main CNN backbone because its dense connectivity structure allows for efficient feature reuse. The architecture includes four dense blocks with a growth rate k of 32 and generates feature maps with channel sizes of 256, 512, 1280, and 1664 from stage 1 to stage 4, respectively. Within a dense block, each layer receives the feature maps of all preceding layers as input. This dense connectivity structure facilitates gradient flow and enhances feature propagation. The output feature map $F_{l}$ for the l-th layer within a dense block is calculated as shown in Equation (1).

(1) $$F_{l}=H_{l}([F_{0},F_{1},\ldots ,F_{l-1}])$$

where $\left[F_{0},F_{1},\ldots ,F_{l-1}\right]$ denotes the concatenation of feature maps from all previous layers, and $H_{l}$ represents the composite function consisting of Batch Normalization, ReLU activation, and 3 × 3 convolution. This dense connectivity structure significantly reduces the number of parameters while increasing feature reuse. Thus, the network becomes both parameter-efficient and more effectively captures subtle retinal structures, which are critical for ERM classification.

Transition layers located between dense blocks first reduce channel size with 1 × 1 convolution, then perform down sampling with 2 × 2 average pooling. Thus, while the number of channels increases, the spatial resolution gradually decreases from 56 × 56 Stage 1 to 7 × 7 Stage 4. We extract intermediate features not only from the last layer but from all four stages. In this way, we preserve information at different scales and it becomes easier to capture pathological changes observed at different spatial scales. The final dense block output produces a feature tensor $F_{dense}^{final}\in R^{7\times 7\times 1664}$ that encodes rich semantic information about retinal morphology.

### 2.4. Vision Transformer Branch for Global Context Modeling

To complement the local receptive field structure of the CNNs, we add a Vision Transformer ViT-B/16 branch that captures global contextual relationships throughout the entire OCT scan. The input image is divided into N 196 non-overlapping patches of 16 × 16-pixel size. Each patch is then transferred to a 768-dimensional embedding space via a linear transformation as given in Equation (2).

(2) $$E_{0}=[x_{cls};x_{1}^{p}W_{p};x_{2}^{p}W_{p};\ldots ;x_{N}^{p}W_{p}]+E_{pos}$$

where $x_{i}^{p\;}$ represents the *i*-th flattened patch, $W_{p}\in R^{\left(16\times 16\times 3\right)\times 768}\;$ is the learnable projection matrix, $x_{cls}\;$ is a learnable class token prepended to the sequence, and $E_{pos}\;$ denotes fixed positional embeddings that preserve spatial information. These patch embeddings are then processed by 12 transformer encoder layers. Each encoder layer contains a multi-head self-attention (MHSA) block followed by a feed-forward network (FFN), each wrapped with residual connections and layer normalization, as defined in Equations (3) and (4), respectively.

(3) $$Z_{l}^{\prime }=\mathrm{MHSA}(\mathrm{LN}(Z_{l-1}))+Z_{l-1}$$

(4) $$Z_{l}=\mathrm{FFN}(\mathrm{LN}(Z_{l}^{\prime }))+Z_{l}^{\prime }$$

The multi-head self-attention mechanism allows the model to focus on different spatial regions simultaneously. This captures long-range relationships, and this feature is particularly valuable in identifying subtle retinal distortions seen in early-stage ERM [29,30]. After processing through all transformer layers, we extract the final class token representation $F_{vit}^{cls}\in R^{768}$, which encodes global image-level features informed by all patch interactions.

### 2.5. Novel Feature Processing Modules

In this paper, to effectively leverage the complementary strengths of CNN and transformer capabilities, three dedicated modules are added that enhance and integrate multimodal representations.

#### 2.5.1. Multi-Scale Dense Feature Aggregation (MDFA)

**Figure 3:**
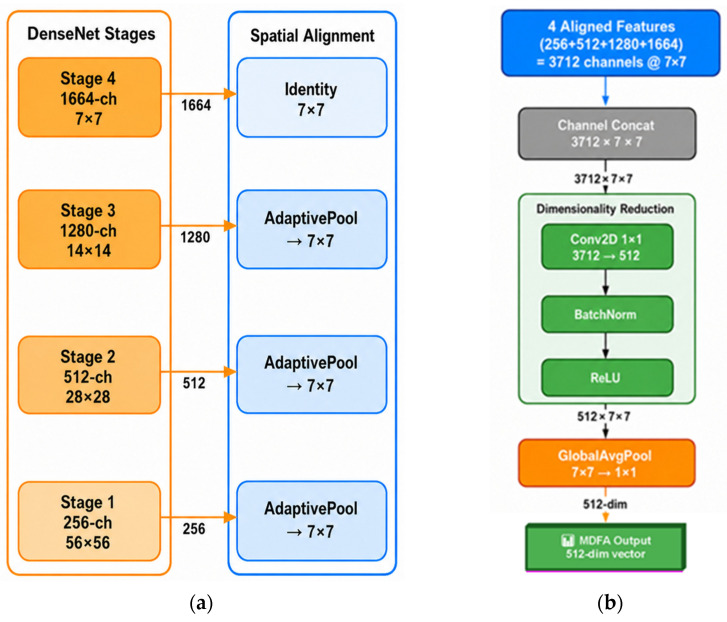
Multi-Scale Dense Feature Aggregation (MDFA) module. (**a**) Feature maps from four DenseNet stages are spatially aligned by adaptive average pooling and projected into a shared channel space using 1 × 1 convolutions. (**b**) The aligned multi-scale features are concatenated and fused to produce a compact representation that retains both low-level structural detail and high-level semantic information.

The MDFA module solves the problem of combining features from different stages of DenseNet, which have different spatial resolutions and levels of meaning, into a single structure. As shown in Figure 3, MDFA uses adaptive spatial alignment to bring features to the same dimensionality and applies channel-based dimensionality reduction to reduce the number of channels. This results in a single, unified multi-scale representation that brings together information across scales. For each DenseNet stage s∈{1,2,3,4} producing feature maps $F_{s}\in R^{H_{s}\times W_{s}\times C_{s}}$, we first apply adaptive average pooling to standardize all feature maps to a common spatial size of 7 × 7 as given in Equation (5).

(5) $$F_{s}^{aligned}=\mathrm{AdaptivePool}(F_{s})\in R^{7\times 7\times C_{s}}$$

More specifically, let F1, F2, F3, and F4 denote the outputs of the four DenseNet stages with heterogeneous spatial dimensions (dim) and channel depths. Adaptive average pooling is applied to each stage output so that all feature tensors are resized to a common spatial resolution of 7 × 7. A 1 × 1 convolution is then used to project each pooled tensor into a shared 512-channel latent space. Formally, the aligned features can be written as $F_{s}^{aligned}=\mathrm{AdaptivePool}\left(F_{s}\right)\in R^{7\times 7\times C_{s}}$ where AAP denotes adaptive average pooling. The aligned tensors are concatenated along the channel dimension and passed through subsequent convolutional fusion layers to obtain a compact multi-scale representation. This design allows low-level structural details and high-level semantic cues to be retained within a unified feature tensor. This spatial alignment ensures that features from early stages (which capture low-level textures and edges) and late stages (which encode high-level semantic patterns) can be meaningfully combined despite their different receptive field sizes [31]. Subsequently, we perform channel-wise dimensionality reduction through 1 × 1 convolutions to compress each stage’s features to a uniform 512-dimensional space, followed by batch normalization and ReLU activation. The aligned and normalized features are then concatenated along the channel dimension and fused through a series of convolutional operations as defined in Equation (6).

(6) $$F_{mdfa}=\mathrm{GlobalAvgPool}({\mathrm{Conv}}_{1\times 1}(\mathrm{Concat}[F_{1}^{aligned},F_{2}^{aligned},F_{3}^{aligned},F_{4}^{aligned}]))$$

This process yields a compact 512-dimensional feature vector $F_{mdfa}\in R^{512}$ that encapsulates multi-scale information from all DenseNet stages, enabling the model to simultaneously consider both fine-grained retinal layer structures and broader morphological patterns.

#### 2.5.2. Adaptive Feature Calibration (AFC)

**Figure 4:**
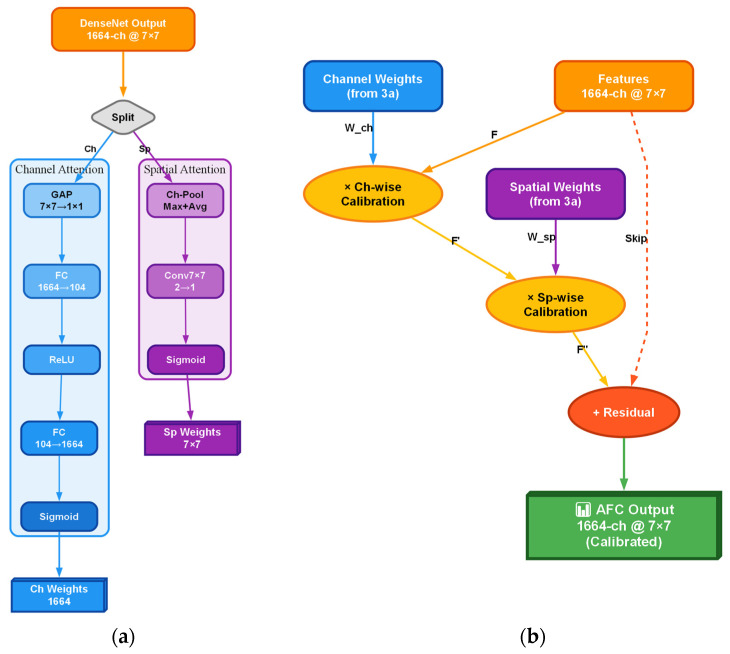
Adaptive Feature Calibration (AFC) module. Abbreviations: Ch, Channel; Sp, Spatial; GAP, Global Average Pooling; FC, Fully Connected. (**a**) Channel attention and spatial attention pathways are computed from the final DenseNet feature map using pooled descriptors and convolutional refinement. (**b**) The recalibrated feature map is obtained through sequential channel-spatial attention with residual preservation of the original representation.

The AFC module enhances the discriminative power of the final DenseNet features through dual-pathway channel and spatial attention mechanisms, as illustrated in Figure 4. Unlike standard attention modules that operate independently, AFC jointly recalibrates features across both channel and spatial dimensions with explicit residual connections to preserve original feature information [32].

Given the final dense block output $F_{dense}^{final}\in R^{7\times 7\times 1664}$, the channel attention pathway aggregates spatial information through both global average pooling and global max pooling, followed by shared multi-layer perceptron’s (MLPs) with a reduction ratio of 16 as shown in Equation (7).

(7) $$M_{c}=\sigma (\mathrm{MLP}(\mathrm{AvgPool}(F_{dense}^{final}))+\mathrm{MLP}(\mathrm{MaxPool}(F_{dense}^{final})))$$

where σ denotes the sigmoid activation function, and $M_{c}\in R^{1\times 1\times 1664}$ represents the learned channel-wise attention weights. This mechanism identifies which feature channels are most relevant for ERM classification.

In parallel, the spatial attention pathway operates on channel-wise aggregated features to determine important spatial locations as shown in Equation (8).

(8) $$M_{s}=\sigma ({\mathrm{Conv}}_{7\times 7}([{\mathrm{AvgPool}}_{c}(F_{dense}^{final});{\mathrm{MaxPool}}_{c}(F_{dense}^{final})]))$$

where $\left[\cdot ;\cdot \right]$ denotes concatenation along the channel dimension, and $M_{s}\in R^{7\times 7\times 1}$ provides spatial attention weights that highlight anatomically relevant regions such as the foveal area and retinal layer interfaces.

The final calibrated features are obtained through sequential application of both attention mechanisms with residual connections as given in Equation (9)

(9) $$F_{afc}=F_{dense}^{final}⊙M_{c}⊙M_{s}+F_{dense}^{final}$$

where ⊙ denotes element-wise multiplication. The residual connection ensures gradient stability and prevents over-suppression of features, while the dual attention mechanism adaptively emphasizes diagnostically relevant patterns. The use of dual-pathway attention in AFC was motivated by the fact that ERM-related abnormalities are selective both in terms of feature channels and anatomical location. Channel attention helps emphasize diagnostically informative representation maps, while spatial attention highlights localized retinal regions such as the fovea and vitreoretinal interface. Their sequential combination therefore provides a principled mechanism for suppressing irrelevant responses while preserving clinically meaningful structures.

#### 2.5.3. Cross-Attention Feature Fusion (CAFF)

**Figure 5:**
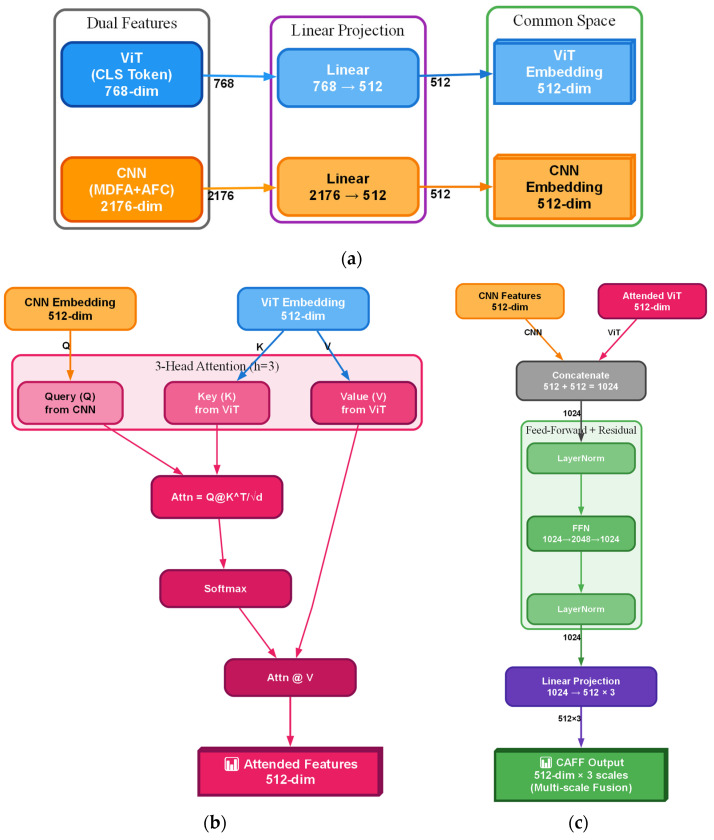
Cross-Attention Feature Fusion (CAFF) module. (**a**) CNN and transformer features are projected into a common embedding space. For the transformer branch, the CLS (Classification) token—the learnable token in ViT that aggregates global image information—is used as the feature representation (**b**) Bidirectional cross-attention enables each feature stream to attend (Attn) to complementary information from the other modality. (**c**) The attended features are concatenated and refined through a fusion MLP to obtain the final multimodal representation.

The CAFF module performs sophisticated fusion of CNN and transformer features through a cross-attention mechanism that allows each modality to selectively attend relevant information from the other [33]. As shown in Figure 5, CAFF first projects both feature streams into a common 512-dimensional embedding space to facilitate interaction.

The DenseNet features (after MDFA and AFC processing) and ViT class token are independently projected: $F_{cnn}^{embed}=W_{cnn}F_{cnn}$ and $F_{vit}^{embed}=W_{vit}F_{vit}^{cls}$, where $W_{cnn},\;W_{vit}\in R^{512\times d_{in}}$ are learnable projection matrices. These embeddings serve as queries, keys, and values in a cross-attention mechanism as given as Equation (10).

(10) $$\mathrm{Attention}(Q,K,V)=\mathrm{softmax}\left(\frac{QK^{T}}{\sqrt{d_{k}}}\right)V$$

Specifically, CNN features attend to transformer features and vice versa: $F_{cnn\to vit}=\mathrm{Attention}(F_{cnn}^{embed},F_{vit}^{embed},F_{vit}^{embed})$ captures global context relevant to local CNN features, while $F_{vit\to cnn}=\mathrm{Attention}(F_{vit}^{embed},F_{cnn}^{embed},F_{cnn}^{embed})$ grounds transformer representations with detailed local information.

The attended features are concatenated and passed through a feed-forward fusion network consisting of two linear layers with LayerNorm and residual connections as shown in Equation (11).

(11) $$F_{caff}=\mathrm{Linear}(\mathrm{LayerNorm}(\mathrm{Linear}([F_{cnn\to vit};F_{vit\to cnn}])))\in R^{512\times 3}$$

This cross-modal fusion enables the model to leverage both the precise anatomical localization capabilities of CNNs and the holistic contextual understanding of transformers, resulting in a rich multi-scale representation that captures both local pathological details and global retinal morphology patterns. The CAFF design was chosen to enable bidirectional interaction rather than simple concatenation-based fusion. In ERM grading, local structural distortions should inform the interpretation of global morphology, while global retinal context should also refine local feature saliency. Cross-attention provides a principled way to model this mutual dependency between CNN-derived and transformer-derived representations.

### 2.6. Multi-Branch Classification and Deep Supervision

Rather than relying solely on the fused features for classification, our architecture employs a multi-branch classification strategy with deep supervision [34] to enhance learning efficiency and prevent overfitting. As shown in Figure 2, we maintain three parallel classification heads:

Main Classifier: Operates on the fused CAFF features and consists of a 2-layer MLP with dropout (*p* = 0.5) for regularization, producing the primary class predictions $y_{main}$.

Auxiliary DenseNet Classifier: Directly processes the calibrated DenseNet features from AFC, providing supervision signal specifically for the CNN pathway and producing predictions $y_{aux-D}$.

Auxiliary ViT Classifier: Works on the ViT class token features, ensuring the transformer branch learns discriminative representations independently, yielding predictions $y_{aux-V}$.

Each classifier outputs logits for the four ERM classes, which are converted to probability distributions through softmax activation. During training, we employ a weighted deep supervision loss function that combines cross-entropy losses from all three branches as defined in Equation (12).

(12) $$L_{total}=0.7\cdot L_{CE}(y_{main},y_{true})+0.15\cdot L_{CE}(y_{aux-D},y_{true})+0.15\cdot L_{CE}(y_{aux-V},y_{true})$$

where $y_{true}$ represents the ground truth labels, and $L_{CE}$ denotes the standard cross-entropy loss. The weighting scheme (0.7:0.15:0.15) prioritizes the main fused classifier while providing auxiliary gradients to guide individual pathway learning. This deep supervision strategy accelerates convergence, improves gradient flow through deep networks, and acts as an implicit regularizer by preventing over-reliance on a single feature representation.

During inference, only the main classifier’s predictions are used, ensuring that the model benefits from the comprehensive multi-modal fusion without additional computational overhead.

### 2.7. Training Strategy and Hyperparameters

We used the PyTorch 2.0 library to implement the architecture of the model. To accelerate the training of the model and improve the generalization of the learned features for the medical image data set, we employed the technique of transfer learning by setting the initial state of the DenseNet121 backbone as well as the ViT-B/16 branch to the pre-trained weights obtained for the ImageNet data set. To train the model, we employed the AdamW [35] optimizer with an initial learning rate of 3 × 10^−4^ and a weight decay of 1 × 10^−4^ to prevent the occurrence of overfitting. To assist the model in climbing out of the local minima and converging to the global minimum, we employed the cosine annealing learning rate schedule [36] with warm restarts. The training was carried out for 50 epochs with a batch size (BS) of 32. To prevent the occurrence of overfitting, we employed early stopping with a patience of 10 epochs. As discussed earlier in Section 2.6, we employed the technique of deep supervision by using a weighted loss of 0.7:0.15:0.15 for the three classification branches. To prevent the occurrence of overconfident decisions by the classifier, we employed the technique of label smoothing [37] with epsilon = 0.1. In all classifier heads, dropout with a probability of 0.5 was employed. Batch normalization was used in the convolutional refinement stages of the MDFA module and after the 1 × 1 channel-reduction convolutions, whereas layer normalization was retained within the transformer branch and the CAFF MLP. The best-performing model was selected for evaluation with the test set. To ensure reproducibility of the results, fixed random seeds were employed for all the experiments.

Hyperparameter values were selected through controlled validation experiments on the internal validation split. In particular, we compared a small set of candidate learning rates, weight decay values, dropout probabilities, and loss-weight configurations, and selected the final setting based on validation accuracy and macro-F1 stability. Rather than performing an exhaustive search, we adopted a constrained tuning strategy to balance reproducibility and computational feasibility.

## 3. Experimental Results

### 3.1. Evaluation Metrics

To evaluate the performance of the model, we have employed a range of metrics that are suitable for multi-class classification in medical image analysis. For instance, accuracy measures the overall correctness of the model for multi-class classification. Precision, recall, and F1-score were also evaluated in both macro and weighted versions to measure the performance of the model in multi-class classification. In addition, the model’s performance is evaluated using Cohen’s Kappa (κ) statistic, which is helpful in multi-class classification when the classes are imbalanced [38]. The Matthews Correlation Coefficient is also employed in multi-class classification, which is informative even when the classes are imbalanced. Finally, the Area Under the Receiver Operating Characteristic Curve is also evaluated in both macro and micro versions in a one-vs-rest manner to measure the performance of the model in multi-class classification in terms of distinguishing between severity levels [39].

### 3.2. Comparative Performance Analysis

To establish a fair comparison between our DenseViT-OCT architecture and other models, we conducted an extensive set of head-to-head evaluations against 19 strong deep learning architectures. These architectures span a broad range of design approaches and philosophies. We tested our model against classic CNNs such as VGG16, VGG19, and ResNet18. Additionally, our model was evaluated against densely connected networks such as DenseNet121. We also compared against highly efficient architectures such as EfficientNet (B0, B3, B4) [40], as well as lightweight networks such as SqueezeNet and ShuffleNetV2 [41]. Moreover, our model was evaluated against modern CNNs such as ConvNeXt-Tiny/Small [42], RegNetY-400MF [43], and MaxViT-T. Our model was also tested against pure transformer networks [44] such as ViT-B/16, ViT-B/32, Swin-T, and Swin-S. Furthermore, our model was evaluated against inception-based networks such as GoogLeNet [45]. To ensure a fair comparison, all models were trained under identical conditions.

Table 2 reports the performance results for all 20 models evaluated in our experiments. DenseViT-OCT achieved the best single-run performance on the internal test set, with an accuracy of 94.76%, a macro-F1 of 93.47%, and a Cohen’s kappa of 92.62%. The strongest competing baseline was ConvNeXt-Small, which achieved 94.08% accuracy and 92.74% macro-F1. Although the margin over the best baseline is modest in single-run comparison, subsequent ablation, repeated multi-run evaluation, and external validation analyses provide additional evidence regarding the robustness and contribution of the proposed design.

From the baseline models, modern CNNs such as ConvNeXt-Small, EfficientNet-B3, and MaxViT-T have recorded 94.08%, 93.85%, and 93.62%, respectively. Older architectures such as ResNet18, DenseNet121, and VGG have also recorded decent results in the 92–93% range. These results confirm their continued relevance to solving medical image classification problems. Transformer models have recorded varied results, with ViT-B/16 achieving 91.57%, whereas a less accurate variant, ViT-B/32, recorded a showed substantially lower performance, achieving 55.13%, most likely as a result of over-sampling that destroyed finer retinal details necessary for ERM assessment. Lightweight mobile models such as MobileNetV2/V3 and ShuffleNetV2 have recorded decent though less accurate results compared to their counterparts. This suggests that model capacity is essential for solving such a difficult classification problem.

### 3.3. Ablation Study

To quantify the contribution of each architectural component, we performed an ablation study by progressively removing MDFA, AFC, CAFF, and deep supervision from the full DenseViT-OCT framework. As shown in Table 3, the complete model achieved the best overall performance. Removing MDFA, AFC, or CAFF led to small but consistent reductions in accuracy, macro-recall, macro-F1, and Cohen’s kappa, indicating that each proposed module contributed positively to the final representation quality. The largest performance drop was observed when deep supervision was removed, reducing accuracy from 94.76% to 93.62% and macro-F1 from 93.47% to 92.19%, suggesting that auxiliary supervision played a substantial role in stabilizing optimization and improving discriminative learning. In addition, the hybrid variant without MDFA, AFC, and CAFF remained inferior to the full model, while standalone DenseNet121 and ViT-B/16 also underperformed the proposed architecture. These findings support both the complementary value of hybrid local-global modeling and the incremental contribution of the proposed custom modules.

### 3.4. Multi-Run Statistical Robustness

Because the single-run accuracy margin over the strongest baseline was relatively small, we further evaluated robustness across five random seeds while keeping the patient-level data split fixed. Table 4 summarizes the distribution of performance across repeated runs. DenseViT-OCT achieved the best average results, with an accuracy of 0.9399 ± 0.0052, a macro-F1 score of 0.9264 ± 0.0061, and an AUC of 0.9914 ± 0.0015. In comparison, ConvNeXt-Small achieved 0.9312 ± 0.0037 accuracy, 0.9172 ± 0.0041 macro-F1, and 0.9910 ± 0.0005 AUC. The bootstrap 95% confidence interval for DenseViT-OCT mean accuracy was 0.9303–0.9494, compared with 0.9198–0.9417 for ConvNeXt-Small.

To further assess whether these differences were statistically meaningful, we performed pairwise statistical comparisons between DenseViT-OCT and the strongest representative baselines. As shown in Table 5, paired *t*-tests on seed-wise accuracies showed statistically significant improvements of DenseViT-OCT over ConvNeXt-Small (*p* = 0.0066), DenseNet121 (*p* = 0.0479), and ViT-B/16 (*p* = 0.0058), with large effect sizes in all cases. However, McNemar analysis on the fixed test set did not reveal a significant instance-level difference between DenseViT-OCT and ConvNeXt-Small, indicating that the proposed model provides a more favorable average performance across repeated runs while preserving partially overlapping sample-wise error patterns with the strongest baseline. We therefore interpret the observed performance gain as robust but moderate rather than absolute.

### 3.5. External Validation on the OCTDL Dataset

To assess external generalization, we evaluated the trained DenseViT-OCT model on the public OCTDL dataset [46], which consists of ERM and normal OCT images collected from multiple centers. Since a publicly available external dataset with four-class ERM severity labels was not available, this experiment was formulated as a binary classification (ERM vs. Normal). This was achieved by mapping the predicted classes {Stage 1, Stage 2, Stage 3} to the “ERM” category and the {Normal} class to the “Normal” category.

As detailed in Table 6, DenseViT-OCT achieved 90.76% accuracy, 95.48% sensitivity, 88.55% specificity, and an AUC of 97.61% on this multicenter dataset. These findings suggest that the model retains strong ERM detection capability beyond the development cohort. However, since the external labels were binary rather than stage-specific, these results should be interpreted as an external validation of ERM detection generalization rather than a full validation of the four-class severity grading system.

In addition, the model achieved a PPV of 79.57%, an NPV of 97.67%, an F1-score of 86.80%, Cohen’s kappa of 0.7978, and a Matthews correlation coefficient (MCC) [38,39] of 0.8057. The high Negative Predictive Value (NPV) indicates that images predicted as normal were rarely misclassified as ERM cases, while the MCC and Cohen’s kappa values suggest substantial agreement and robust binary classification performance. The confusion matrix values were 148 true positives, 294 true negatives, 38 false positives, and 7 false negatives.

### 3.6. Detailed Classification Performance

The proposed DenseViT-OCT model reported comprehensive performance metrics that reflected high accuracy and balance in all severity classes. The overall test set accuracy reported was 94.76%. The precision reported was 93.76%, and the overall recall reported was 93.22%. The F1 score reported was 93.47%. The weighted precision reported was 94.76%, and the overall weighted recall reported was 94.76%. The weighted F1 score reported was 94.74%. This indicates that the model performed consistently despite the moderate level of class imbalance in the dataset. The Cohen’s Kappa score reported was 92.62%. This indicates that the model performed almost perfectly. The Matthews correlation coefficient reported was 92.63%. This indicates that the model is reliable when all elements in the confusion matrix are considered. The discriminative power of the model was validated by reporting 98.95% in macro AUC and 99.09% in micro AUC.

The detailed results for each class in the classification task are given in Table 7, showing the model’s power in discriminating between normal retinal anatomy and the different levels of ERM severity. For the Normal OCT class, the results were exceptional, with precision at 98%, perfect recall at 100%, and an F1-score of 99% on 184 test samples. For the ERM severity classes, the best-performing class was Stage 3, or Severe, with the highest F1-score at 95%, precision at 95%, and recall at 94% on 85 test samples, showing the dominant morphological characteristics in this stage. For Stage 1, or Mild, the results were strong, with an F1-score of 91%, precision at 94%, and recall at 89% on 81 test samples, while the results for Stage 2, or Moderate, were lower, with an F1-score of 89%, precision at 88%, and recall at 90% on 89 test samples. The lower results for Stage 2 than Stage 3 are due to the inherent difficulties in the manifestations of this stage, which show less pronounced morphological characteristics than the severe stage but not the definite anatomical boundaries seen in the mild stage or normal tissue.

### 3.7. Confusion Matrix and Error Pattern Analysis

**Figure 6:**
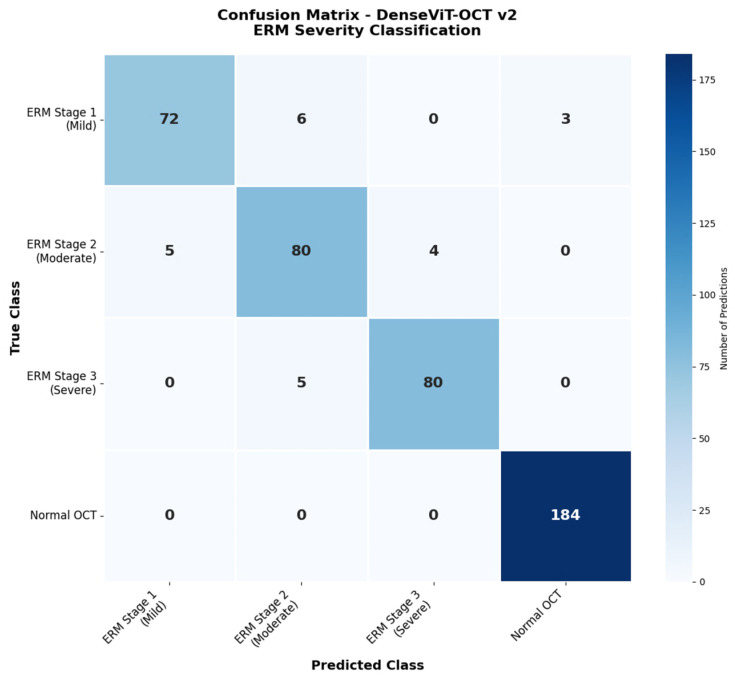
Confusion matrix of DenseViT-OCT on the internal four-class test set. The matrix shows strong diagonal dominance, with most errors occurring only between adjacent ERM severity stages rather than between clinically distant categories.

The confusion matrix presented in Figure 6 provides detailed insights into the classification patterns and error distribution across all four classes. The strong diagonal dominance throughout the matrix confirms robust classification performance, with the majority of predictions correctly aligned with ground truth labels. Among the 439 test samples, the model achieved correct classification for 416 cases, resulting in an overall accuracy of 94.76%.

Examining the classification patterns for each class reveals interpretable error distributions. For ERM Stage 1 cases, the model correctly classified 72 out of 81 samples, with 6 cases misclassified as Stage 2 and 3 cases as Normal. This error pattern reflects the diagnostic challenge of distinguishing minimal membrane presence from normal anatomical variations and early disease progression. ERM Stage 2 cases showed 80 correct classifications out of 89 total samples, with 5 cases confused with Stage 1 and 4 cases with Stage 3, demonstrating the difficulty of precisely delineating moderate severity that shares features with both adjacent categories. Stage 3 cases exhibited 80 correct classifications from 85 samples, with all 5 misclassifications occurring as Stage 2, indicating that errors involving severe disease consistently involved confusion with the immediately preceding severity level rather than more dramatic misclassifications. Most remarkably, all 184 Normal OCT cases were correctly identified without any false negatives, demonstrating perfect sensitivity for healthy retinal tissue detection.

A critical finding from the confusion matrix is the complete absence of misclassifications between non-adjacent severity categories. No Stage 1 cases were misclassified as Stage 3, no Stage 3 cases were confused with Stage 1 or Normal, and no ERM cases of any severity were misclassified as Normal (with the exception of the 3 Stage 1 cases representing minimal disease). This graduated error pattern, where misclassifications occur exclusively between adjacent severity levels, suggests that the model has learned a coherent representation of disease progression rather than relying on spurious correlations or dataset-specific artifacts.

### 3.8. Computational Cost Analysis

To complement the predictive performance analysis, we evaluated the computational footprint of the proposed architecture alongside several representative comparison models. As summarized in Table 8, DenseViT-OCT contains 107.25 million trainable parameters and requires 14.737G Multiply–Accumulate Operations (MACs), with an average inference latency of 17.78 ms per image.

Compared with the strongest baseline, ConvNeXt-Small, the proposed model is computationally heavier (107.25M vs. 49.46M parameters; 17.78 ms vs. 5.93 ms latency), reflecting the additional cost of dual-stream processing and dedicated fusion modules. However, the full model still supports real-time inference at 56.2 FPS for batch size 1 and 410.5 FPS for batch size 16, indicating that the added complexity remains practical for clinical decision-support settings. Ablation variants further demonstrate that the MDFA, AFC, and CAFF modules introduce only modest incremental overhead relative to the hybrid backbone.

All measurements were obtained on an NVIDIA GeForce RTX 4080 SUPER GPU using PyTorch 2.7.1 with CUDA 11.8. Latency was calculated as the average per-image inference time, and throughput is reported as frames per second (FPS) for batch sizes of 1 and 16.

### 3.9. Model Interpretability Through Visual Explanations

To further visualize the decision-making process of the proposed DenseViT-OCT model, we have employed two different explainable approaches, namely Gradient-weighted Class Activation Mapping (Grad-CAM++) [47] and Local Interpretable Model-agnostic Explanations (LIME) [48]. These visualization techniques help to better understand the regions of the OCT images that are more influential in the decision-making process of the DenseViT-OCT, thus indicating the presence of clinically relevant anatomical features in the images.

**Figure 7:**
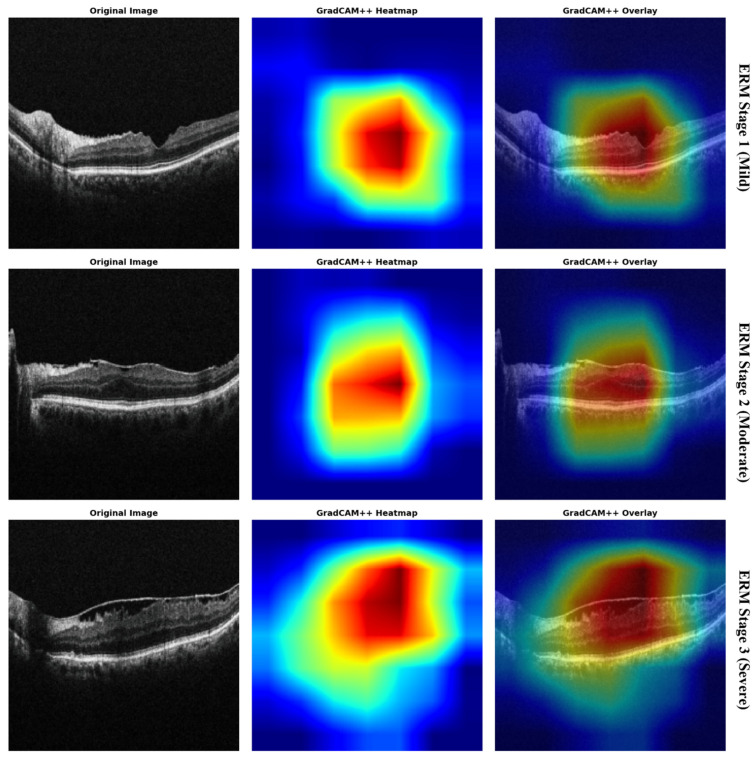
Grad-CAM++ visualizations for representative ERM severity cases. Each row corresponds to one severity stage, and the columns show the original OCT image, the activation heatmap, and the overlay. Warmer colors indicate regions that contributed more strongly to the predicted class.

In Figure 7, the Grad-CAM++ heat maps are provided for the representative cases of each of the different ERM severity stages. The Grad-CAM++ technique generates class-discriminative localization maps by computing the gradient-weighted summation of the feature maps in the last convolutional layers of the DenseNet121 backbone, with warmer colors (red, orange) indicating regions of higher importance for the predicted class. In the case of the ERM Mild class, the heat maps show the activation regions concentrated around the foveal region and the inner retinal layers, with a relatively broad spatial distribution due to the diffuse and mild formation of the ERM in the early stages. The Moderate class shows more intense activation regions at the foveal center and regions of visible contour distortion, thus indicating the increased confidence of the DenseViT-OCT in the presence of more pronounced pathological features. The heat maps for the ERM Severe class show the highest activation intensity, with sharply defined activation peaks at the regions of prominent membrane formation and retinal folding, along with the presence of secondary features such as intraretinal cystic changes and disrupted layer boundaries.

**Figure 8:**
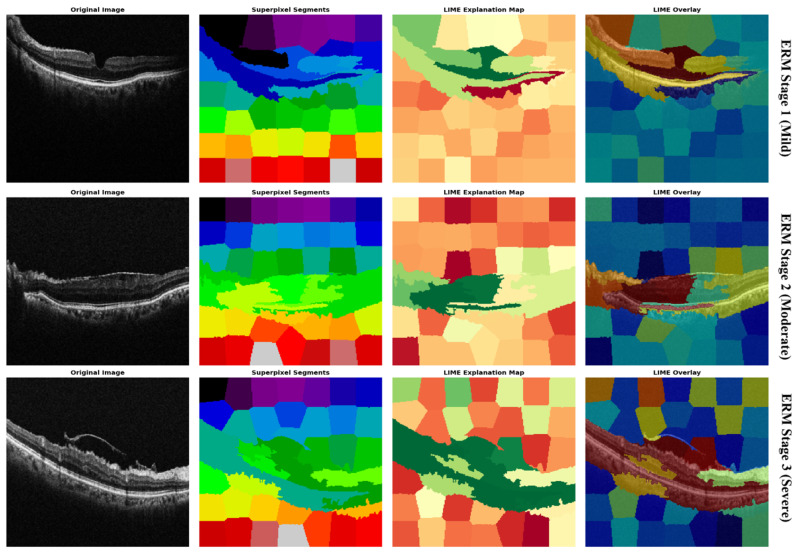
LIME-based explanations for representative ERM severity cases. For each example, the panels show the original OCT image, superpixel segmentation, the explanation map, and the overlay. Green regions indicate positive contribution to the predicted class.

Figure 8 demonstrates LIME-based superpixel explanations for representative cases as presented in Figure 7. An alternative view for interpreting the model is provided by LIME by perturbing segments of the superpixel and determining their contribution to the probability predictions. Each visualization is composed of four sections: the original OCT image, the OCT image segmented into perceptually meaningful regions called superpixels via SLIC segmentation, a color-coded explanation map where green represents positive contribution to the predicted class, and a composite image comprising the explanation map and original OCT image for anatomical reference. In Stage 1 cases, there are multiple superpixels in the foveal region that have a positive contribution to the mild ERM classification, with a high level of importance given to areas at the vitreoretinal interface. In Stage 2 cases, there is a high level of importance given to areas overlapping visible membrane structures and foveal contour irregularities, as well as peripheral areas that have a moderate level of importance. In Stage 3 cases, there is a high level of positive contribution from areas overlapping prominent retinal folds and membrane structures, as well as secondary areas such as retinal layer compression and intra-retinal fluid accumulation.

In both visualization approaches, the model’s attention is consistently drawn to relevant anatomical structures, especially the foveal region and vitreoretinal interface, with minimal activation in irrelevant diagnostic areas like choroidal regions or image borders. The agreement between Grad-CAM++ and LIME in focusing attention on similar relevant anatomical regions using different approaches lends support to the legitimacy of the model’s predictions rather than spurious correlations or dataset-specific effects. The increase in activation strength and localization with disease severity in both Grad-CAM++ and LIME parallels the natural progression of disease severity in clinical evaluation, where more severe disease states exhibit more prominent and localized morphological changes.

## 4. Discussion

### 4.1. Superior Performance Through Hybrid Architecture

This study has shown the effectiveness of hybrid CNN-Transformer architectures in addressing the multi-scale and context-dependent characteristics of ERM severity assessment. The DenseViT-OCT model has achieved the best performance with 94.76% accuracy, along with balanced precision and recall of 93.76% and 93.22%, respectively, outperforming 19 advanced deep learning techniques. The high performance of the DenseViT-OCT can be attributed to the complementary advantages of CNN and Transformer representations. The assessment of ERM severity requires the simultaneous recognition of microstructures and morphological distortions on the retina. The DenseNet branch can effectively recognize local anatomical abnormalities, including irregularities in the retinal layers and membrane formation, whereas the Transformer branch can capture the long-range spatial correlations and global context. The two-stream representation can alleviate the limitations of using CNN alone, which may fail to capture the global context, and using the Transformer alone, which may fail to capture the local microstructures.

The advanced feature processing modules used in the DenseViT-OCT have further contributed to the improvement in the quality of the representations. The MDFA module can effectively pool the multi-scale features, allowing the model to capture the textures and semantic patterns in the images. The AFC module can further improve the discriminative ability of the representations by enhancing the importance of the channels and spatial regions of interest, as well as suppressing the noise. The CAFF module can facilitate the interaction between the CNN and Transformer representations, allowing the representations to be more enriched.

The ablation analysis further clarified the source of this performance. Removing MDFA, AFC, or CAFF led to consistent but relatively modest declines, indicating that each module contributes incrementally to representation quality. The removal of deep supervision caused the largest degradation, highlighting its importance for optimization stability and branch-level discriminative learning. In repeated experiments across five random seeds, DenseViT-OCT also showed the best average performance, suggesting that the advantage of the proposed architecture is not limited to a single favorable initialization. Taken together, these findings indicate that the model’s gain arises not from one isolated design choice, but from the combined effect of hybrid local-global representation learning, targeted feature refinement, and training stabilization.

### 4.2. Clinical Relevance and Interpretability

From a clinical workflow perspective, the proposed framework may be most useful as a decision-support system rather than a replacement for specialist judgment. In routine practice, OCT-based ERM assessment is used to support follow-up scheduling, monitor structural progression, and inform referral for possible surgical intervention. An automated model that highlights relevant retinal regions while providing stage-consistent predictions may reduce inter-observer variability and improve standardization, particularly in high-volume clinical settings or screening-oriented workflows. Apart from its quantitative performance, DenseViT-OCT has several features that suggest potential clinical relevance as a preliminary decision-support approach, although further prospective validation is required before routine clinical use. DenseViT-OCT was able to attain 100% recall on the Normal OCT category, which is very important to ensure that no healthy patient is misclassified as having the disease. This is especially important in the context of screening, which is very crucial in medicine. From the error analysis, it was observed that DenseViT-OCT was making errors between adjacent stages in the severity of the disease. There were no instances of false classification between the extremes of the disease. This is very similar to the behavior of human clinicians in the field of medicine. Even experienced clinicians can be expected to exhibit similar behavior in the context of diseases that exist on a continuum. The absence of false negatives in this internal test setting is encouraging; however, this finding should be confirmed in larger external and prospective cohorts before drawing conclusions about clinical safety.

The interpretability results obtained from the application of Grad-CAM++ and LIME were consistent in pointing to the anatomical regions of interest in the OCT images. The model was especially interested in the foveal region and the vitreoretinal interface. These results were consistent across the two interpretability tools that were employed. The increase in intensity of the activation across the stages of the disease is similar to the grading of the disease in the context of its severity.

In addition to the aforementioned built-in interpretation capabilities, the clinical application value of DenseViT-OCT becomes further substantiated through the latest evidence regarding AI-enhanced ERMs diagnostics. Jin et al. [15] have shown that it is feasible for an explainable neural network architecture to attain accurate classification of ERMs with a cross-device cross-center test performance. On the contrary, Sato & Kuramoto [49] have shown that a general language model (ChatGPT-4o) reached only a moderate degree of consistency with ophthalmologist diagnoses for Govetto staging of ERMs with an initial detection rate of only 26.4%, which means that specialized AI architectures are necessary for reliable diagnostics. Further supporting this conclusion is a study comparing the performance of automated machine learning and expert-made algorithms designed for the diagnosis of vitreomacular interface disorders [50]. According to the results, neural networks designed by experts have consistently proven to be more efficient than automated AutoML architectures, especially when dealing with cases that are difficult to diagnose. Based on the evidence provided, it can be said that DenseViT-OCT, an algorithm with a specific design, is a promising candidate for a clinically applicable diagnostic tool.

### 4.3. Comparison with Related Work and Broader Implications

A direct comparison with prior ERM classification studies is presented in Table 9, highlighting differences in dataset scale, task complexity, validation strategies, and reported performance.

Most importantly, performance is best understood in relation to task complexity, as multi-class severity grading is significantly more complex than the binary classification of ERM and DME detection. Although other studies have reported higher accuracy in their results, these are typically for simpler classification tasks than those faced in this paper. Our model, meanwhile, is designed to deal with a four-class severity grading task that is closer to real-world clinical decision-making scenarios.

In comparison to other multi-class studies that have reported accuracy between 79.4% and 87.8%, DenseViT-OCT demonstrates significant performance improvements while using strict patient-level data splitting to avoid data leakage in validation sets, which is a more realistic approach than image-level data splitting. Another factor that plays an important role is the composition of the dataset that is being used in the study. It is possible that studies that have fewer patients in their dataset may have higher risks of overfitting despite high accuracy results. Our dataset, meanwhile, contains 2195 OCT images from 397 patients and provides a good balance between dataset diversity and annotation quality. Another significant advantage of DenseViT-OCT is that it provides in-depth analysis in terms of interpretability, which is also an important consideration in clinical settings for establishing trust in machine learning predictions. These results suggest that the use of a hybrid approach that combines both local and global feature extraction could potentially be an important direction in the development of OCT-based diagnostic systems for complex macular diseases.

In addition, the external validation experiment on the public multicenter OCTDL dataset provides preliminary evidence that the learned representation generalizes beyond the internal single center cohort. Importantly, because OCTDL only supported binary ERM-versus-normal evaluation, this result should not be interpreted as full external validation of stage-wise severity grading. Nevertheless, the strong external AUC suggests that the model captures clinically meaningful ERM-related patterns that are not restricted to the original dataset.

### 4.4. Limitations and Considerations

Several limitations of this study should be acknowledged. First, the primary development dataset was collected retrospectively from a single institution. Although we performed strict patient-level splitting for internal evaluation and added an external validation experiment on the public multicenter OCTDL dataset, the latter could only be conducted at the binary ERM-versus-normal level because a publicly available external dataset with compatible four-class ERM severity labels was not available. Therefore, full multicenter external validation of stage-wise ERM grading remains an important next step before broader clinical generalization can be claimed.

Second, the proposed framework operates on individual 2D OCT B-scans rather than complete OCT volumes or patient-level aggregated predictions. While this design simplifies model development and allows slice-level analysis, it does not fully reflect routine clinical assessment, where ERM severity is typically interpreted across multiple neighbouring scans and in conjunction with volumetric context. Accordingly, the present system should be viewed as a B-scan-level decision-support framework rather than a complete patient-level grading solution.

Third, the ground-truth labels were assigned retrospectively by a single ophthalmologist with more than 15 years of experience, using Govetto-inspired OCT staging criteria adapted to the four-class setting of this study. Although this ensured clinically informed labeling, inter-observer agreement could not be assessed. This limitation is particularly relevant for borderline cases between adjacent severity stages, where labeling uncertainty may influence model training and evaluation.

Fourth, the images were resized to 224 × 224 pixels to match the input requirements of the selected backbones. This standardization may reduce fine anatomical detail and could partly affect subtle-stage discrimination. In addition, the model was trained using imaging data alone and did not incorporate complementary clinical variables such as visual acuity, symptom duration, or demographic information, which may further improve performance in challenging cases.

Finally, DenseViT-OCT achieved improved predictive performance at the cost of increased computational complexity relative to lighter baselines such as ConvNeXt-Small. Although the measured inference speed remained practically usable on modern GPU hardware, this higher computational footprint may limit direct deployment in low-resource environments and should be considered in future deployment-oriented studies.

### 4.5. Future Directions

Several directions may further improve the clinical applicability of this work. First, future studies should perform true multicenter external validation for stage-wise ERM severity grading using independently collected and consistently annotated datasets. Second, the present B-scan-based framework should be extended toward volumetric OCT analysis and patient-level prediction by integrating information across neighbouring slices. Third, multimodal learning strategies that incorporate clinical metadata, such as visual acuity and demographic variables, may improve performance in ambiguous cases. Fourth, future work should investigate uncertainty quantification approaches, including Bayesian deep learning or confidence-aware calibration strategies, so that model predictions can be accompanied by clinically interpretable confidence estimates. Finally, prospective reader-assistance studies would be valuable to determine whether the proposed system improves consistency, speed, or decision quality in real clinical workflows.

## 5. Conclusions

In this study, we proposed DenseViT-OCT, a hybrid CNN-transformer framework for automated ERM severity classification from OCT B-scans. On the internal four-class test set, the model achieved 94.76% accuracy and outperformed 19 benchmark architectures. Additional ablation experiments showed that MDFA, AFC, CAFF, and deep supervision each contributed to the final performance, while repeated multi-run evaluation across five random seeds demonstrated that the proposed model maintained the best average accuracy and macro-F1 among the strongest compared models. External validation on the public multicenter OCTDL dataset further showed promising generalization for binary ERM-versus-normal detection.

Beyond predictive performance, DenseViT-OCT exhibited clinically meaningful behavior: normal cases were identified with perfect recall in the internal test set, misclassifications were largely restricted to adjacent severity stages, and explainability analyses consistently highlighted anatomically relevant retinal regions. These characteristics support the potential utility of the model as a decision-support tool for OCT-based ERM assessment. In light of the growing importance of OCT-based grading for ERM severity for the purposes of medical decision-making, prognostication, and longitudinal evaluation, the use of accurate and intelligible artificial intelligence systems can improve objective retinal evaluations in ophthalmology practice in the coming years.

Nevertheless, these findings should be interpreted cautiously in light of the study limitations, including single center development, B-scan-level inference, absence of inter-observer agreement analysis, and lack of full stage-wise external validation. Overall, the results suggest that hybrid local-global modeling is a promising direction for automated ERM analysis, but further multicenter, volumetric, uncertainty-aware, and prospective clinical studies are required before the method can be considered for routine clinical use.

## Figures and Tables

**Table 1:** Comparing the performance of the most advanced method and the proposed approaches for the ERM classification task.

Author	Number of Patients	Number of OCT Images	Number of Classes	Validation Technique	Classification Accuracy (%)	Explainability
Lo et al. [13]	964	1447 ERM, 2171 Normal Total: 3618	2; Normal, ERM	80% Training, 20% Test	99.7	Grad-CAM
Jin et al. [15]	1046	2606 ERM 1968 Normal Total: 4547	6; Normal, Stage-1 to stage-5 ERM	70% Training, 10% Validation, 20% Test	87.8 79.4	not available
Yan et al. [16]	529	2071 ERM 1882 Normal Total: 3953	6; Normal, stage-1 to stage-5 ERM	92%Training, 8% Test	81.3	not available
Gende et al. [17]	20	809 ERM, 162 Normal Total: 2427	2; Normal, ERM	4-Fold Cross-Validation	91.8	not available
Mora et al. [18]	608	660 ERM 1500 Normal Total: 2160	2; Normal, ERM	10-fold cross-validation	99.7	Grad-CAM
Ayhan et al. [19]	461	7016 ERM, 4045 Normal Total: 11,061	3; Normal, stage-1 to stage-2 ERM	75% Training, 10% Validation, 15% Test	89.33	t-SNE
*Proposed*	397	1275 ERM 920 Normal Total: 2195	4; Normal, stage-1 to stage-3 ERM	60% Train/20% Validation/20% Test	94.76	Grad-CAM++, Lime

**Table 2:** Comprehensive Performance Comparison of Deep Learning Models for ERM Classification.

Model	Accuracy	Precision (Macro)	Precision (Weighted)	Recall (Macro)	Recall (Weighted)	F1-Score (Macro)	F1-Score (Weighted)	Cohen Kappa
DenseViT-OCT	0.9476	0.9376	0.9476	0.9322	0.9476	0.9347	0.9474	0.9262
ConvNeXt-Small	0.9408	0.9314	0.9403	0.9238	0.9408	0.9274	0.9404	0.9288
EfficientNet-B3	0.9385	0.9330	0.9391	0.9214	0.9385	0.9263	0.9381	0.9031
MaxViT-T	0.9362	0.9261	0.9354	0.9176	0.9362	0.9215	0.9355	0.9316
ResNet18	0.9339	0.9192	0.9336	0.9171	0.9339	0.9177	0.9334	0.9259
EfficientNet-B0	0.9339	0.9221	0.9336	0.9179	0.9339	0.9197	0.9336	0.9003
ConvNeXt-Tiny	0.9339	0.9319	0.9366	0.9138	0.9339	0.9207	0.9335	0.9088
DenseNet121	0.9339	0.9217	0.9341	0.9154	0.9339	0.9180	0.9336	0.9231
VGG19	0.9294	0.9235	0.9288	0.9085	0.9294	0.9152	0.9283	0.9259
Swin-T	0.9271	0.9121	0.9270	0.9126	0.9271	0.9123	0.9270	0.9174
RegNetY-400MF	0.9248	0.9104	0.9244	0.9078	0.9248	0.9086	0.9242	0.8974
VGG16	0.9226	0.9099	0.9225	0.9015	0.9226	0.9035	0.9207	0.9174
MobileNetV2	0.9226	0.9072	0.9221	0.9041	0.9226	0.9050	0.9218	0.9088
GoogLeNet	0.9226	0.9135	0.9227	0.8996	0.9226	0.9054	0.9217	0.9145
Swin-S	0.9180	0.9117	0.9192	0.8947	0.9180	0.9016	0.9172	0.9145
MobileNetV3-Large	0.9180	0.9092	0.9171	0.8984	0.9180	0.9035	0.9172	0.9117
ViT-B/16	0.9157	0.9041	0.9148	0.8956	0.9157	0.8995	0.9149	0.9117
ShuffleNetV2	0.9134	0.9044	0.9127	0.8905	0.9134	0.8964	0.9122	0.8803
EfficientNet-B4	0.8998	0.8900	0.9000	0.8712	0.8998	0.8782	0.8979	0.8803
SqueezeNet1.1	0.8793	0.8704	0.8802	0.8545	0.8793	0.8594	0.8774	0.8519
ViT-B/32	0.5513	0.3480	0.4073	0.4347	0.5513	0.3336	0.4240	0.5556

**Table 3:** Ablation study of DenseViT-OCT and comparison with standalone backbones.

Variant	Accuracy	Precision (Macro)	Recall (Macro)	F1-Score (Macro)	F1-Score (Weighted)	Cohen’s Kappa
DenseViT-OCT (Full model)	0.9476	0.9376	0.9322	0.9347	0.9474	0.9262
Full—MDFA	0.9453	0.9399	0.9289	0.9338	0.9450	0.9228
Full—AFC	0.9453	0.9381	0.9289	0.9330	0.9450	0.9229
Full—CAFF	0.9453	0.9364	0.9292	0.9324	0.9451	0.9229
Full—Deep Supervision	0.9362	0.9286	0.9170	0.9219	0.9358	0.9100
Hybrid (no MDFA/AFC/CAFF)	0.9271	0.9143	0.9087	0.9113	0.9265	0.8973
DenseNet121	0.9339	0.9217	0.9154	0.9180	0.9336	0.9231
ViT-B/16	0.9157	0.9041	0.8956	0.8995	0.9149	0.9117

**Table 4:** Comparison of Model Performance (Multi-Run Statistics).

Model	Accuracy (Mean ± Std)	Accuracy 95% CI	Macro-F1 (Mean ± Std)	AUC (Mean ± Std)	Best Accuracy	Worst Accuracy
DenseViT-OCT	0.9399 ± 0.0052	[0.9303, 0.9494]	0.9264 ± 0.0061	0.9914 ± 0.0015	0.9453	0.9317
ConvNeXt-Small	0.9312 ± 0.0037	[0.9198, 0.9417]	0.9172 ± 0.0041	0.9910 ± 0.0005	0.9339	0.9248
DenseNet121	0.9130 ± 0.0192	[0.9007, 0.9244]	0.8948 ± 0.0231	0.9904 ± 0.0013	0.9339	0.8884
ViT-B/16	0.9121 ± 0.0080	[0.9002, 0.9230]	0.8938 ± 0.0089	0.9856 ± 0.0019	0.9226	0.9021

**Table 5:** Pairwise statistical comparison of DenseViT-OCT against representative baselines.

Comparison	Delta Accuracy	Paired *t*-Test (*p*-Value)	Cohen’s d	McNemar (*p*-Value)
DenseViT-OCT vs. ConvNeXt-Small	+0.0087	0.0066	2.3126	0.7981
DenseViT-OCT vs. DenseNet121	+0.0269	0.0479	1.2600	<0.0001
DenseViT-OCT vs. ViT-B/16	+0.0278	0.0058	2.4065	0.0007

**Table 6:** External validation performance of DenseViT-OCT on the public OCTDL dataset (binary ERM vs. Normal).

Metric	Value
Accuracy	0.9076
Sensitivity (Recall)	0.9548
Specificity	0.8855
PPV (Precision)	0.7957
NPV	0.9767
F1-Score	0.8680
AUC	0.9761
Cohen’s Kappa	0.7978
MCC	0.8057
TP/TN	148/294
FP/FN	38/7

**Table 7:** Per-Class Classification Performance.

Class	Precision	Recall	F1-Score	Support
ERM Stage 1 (Mild)	0.94	0.89	0.91	81
ERM Stage 2 (Moderate)	0.88	0.90	0.89	89
ERM Stage 3 (Severe)	0.95	0.94	0.95	85
Normal OCT	0.98	1.00	0.99	184
Accuracy			0.95	439
Macro Average	0.94	0.93	0.93	439
Weighted Average	0.95	0.95	0.95	439

**Table 8:** Computational cost analysis of DenseViT-OCT and selected comparison models.

Model	Params (M)	MACs	Latency (ms)	FPS (BS = 1)	FPS (BS = 16)
DenseViT-OCT (Full Model)	107.25	14.737G	17.78	56.2	410.5
Full—Deep Supervision	107.25	14.737G	17.57	56.9	411.8
Full—AFC	106.72	14.728G	17.65	56.6	413.8
Full—MDFA	104.81	14.733G	17.16	58.3	413.8
Full—CAFF	103.04	14.735G	17.31	57.8	416.3
Hybrid (no MDFA/AFC/CAFF)	99.81	14.721G	16.51	60.6	419.3
ConvNeXt-Small	49.46	8.697G	5.93	168.6	972.6
ViT-B/16 Only	85.80	11.285G	3.36	297.7	609.8
ViT-B/32	87.46	2.951G	2.99	334.2	2238.9
DenseNet121 Only	6.96	2.896G	9.22	108.4	1770.8

**Table 9:** Comparison of the proposed DenseViT-OCT with prior ERM classification studies.

Author	Patients	OCT Images	Classes	Validation	Accuracy (%)	Explainability
Lo et al. [13]	964	3618	2	80/20 split	99.7	Grad-CAM
Jin et al. [15]	1046	4547	6	70/10/20 split	87.8/79.4	Not available
Yan et al. [16]	529	3953	6	92/8 split	81.3	Not available
Gende et al. [17]	20	2427	2	4-fold CV	91.8	Not available
Mora et al. [18]	608	2160	2	10-fold CV	99.7	Grad-CAM
Ayhan et al. [19]	461	11,061	3	75/10/15 split	89.33	t-SNE
DenseViT-OCT (Proposed)	397	2195	4	Patient-level split	94.76	Grad-CAM++, LIME

CV: Cross Validation.

## Data Availability

The dataset used in this study is not publicly available due to privacy/ethical restrictions but is available from the corresponding author upon reasonable request.

## Abbreviations

The following abbreviations are used in this manuscript:

ERM	Epiretinal membrane
OCT	Optical coherence tomography
MDFA	Multi-Scale Dense Feature Aggregation
AFC	Adaptive Feature Calibration
CAFF	Cross-Attention Feature Fusion
PVD	Posterior Vitreous Detachment
Grad-CAM	Gradient-weighted Class Activation Mapping
ViT	Vision Transformer
MHSA	Multi-Head Self Attention
